# Innovations in the provision of psychiatric treatment and rehabilitation assistance to the population in the context of the COVID-19 pandemic and their preliminary results

**DOI:** 10.1192/j.eurpsy.2021.752

**Published:** 2021-08-13

**Authors:** I. Belokrylov, A. Munin

**Affiliations:** 1 Department Of Psychiatry And Medical Psychology, Peoples Friendship University of Russia (RUDN University), Moscow, Russian Federation; 2 Department Of Psychiatry And Medical Psychologi, Peoples Friendship University of Russia (RUDN University), Moscow, Russian Federation

## Abstract

**Introduction:**

Numerous publications highlight the need to modify mental health services in the stressful environment of the COVID-19 pandemic. The psychiatric service of the city of Moscow undertook some reforms in this regard.

**Objectives:**

The measures taken are aimed at reducing the risk of infection of mentally ill patients undergoing treatment in dispensaries and day hospitals, as well as at preventing the exacerbation of psychopathological disorders in patients under the prevailing conditions.

**Methods:**

The frequency of visits to the dispensary by patients in stable condition has been reduced to 1 time per week. At the same time, the frequency of telephone consultations with patients and their relatives increased up to 3 times a week. Face-to-face psychotherapy sessions have been replaced by remote (online) sessions. During the study period, medical documents of 60 patients were studied. The number of hospitalizations of outpatients to the hospital was recorded due to the deterioration of the mental state, as well as in connection with the infection with COVID in the period from 04/15/2020 to 06/09/2020.

**Results:**

There were no hospitalizations due to mental deterioration. According to this indicator, the situation in 2020 turned out to be better than last year for the same period. There were also no cases of hospitalization of outpatients in connection with Covid-19.

**Conclusions:**

The presented results indicate the effectiveness of organizational innovations introduced in Moscow in the provision of outpatient psychiatric care. However, these data need clarification and objective scientific interpretation.

**Conflict of interest:**

No significant relationships.

